# “Climatic determinants” and simple thresholds for dengue early warning in Vietnam: a One Health perspective

**DOI:** 10.1016/j.soh.2026.100159

**Published:** 2026-04-28

**Authors:** Nguyen Khoi Quan, Andrew W. Taylor-Robinson

**Affiliations:** a College of Health Sciences, VinUniversity, Hanoi 100000, Viet Nam; b Johns Hopkins University Bloomberg School of Public Health, Baltimore, MD 21205, USA; c Center for Global Health, Perelman School of Medicine, University of Pennsylvania, Philadelphia, PA 19104, USA

**Keywords:** Climate change, Dengue, Early warning system, Threshold-based prediction, El Niño–Southern oscillation, One Health surveillance, Vietnam

## Abstract

In recent years, dengue transmission in Vietnam has transitioned from predictable seasonality to climate-driven instability due to rising temperatures, urbanization, and hydrological extremes, among other factors. Traditional early warning systems based on case averages are becoming increasingly unreliable because of climate non-stationarity, resulting in both false alarms and missed warnings. This perspective summarizes available research on temperature, drought, rainfall extremes, diurnal temperature range, heatwaves, and El Niño anomalies, highlighting biologically-based thresholds that have been observed to precede outbreaks. We advocate for a pragmatic, hybrid early warning system that combines probabilistic national models with operationally simple meteorological triggers adapted to localized conditions. Integrating One Health factors, such as water storage practices, urban livestock habitats, and climate-sensitive vector control technologies, can facilitate optimal responses. This climate-informed, trigger-based approach helps to provide a realistic dengue control and prevention strategy that navigates low-cost and “no regrets” interventions in the face of accelerated environmental change.

## Graphical abstract

**Figure:**
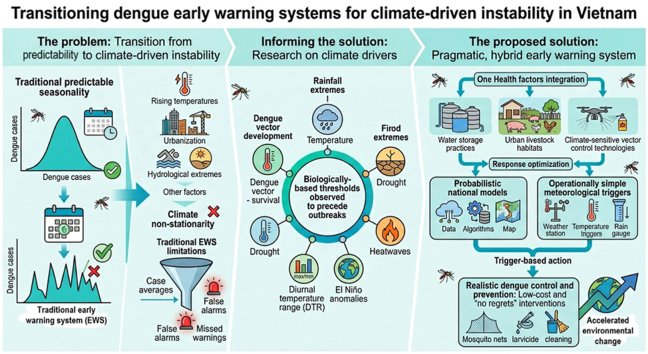

## 1 Introduction

Dengue epidemiology in Vietnam has undergone a significant transition over the last decade, as climatic change, rapid urbanization, and virus evolution have disrupted the typical seasonality that traditionally defined transmission patterns [1,2]. Historically, dengue was concentrated in the tropical southern lowlands, while the north benefited from a thermal “barrier effect” created by cool winters limiting *Aedes* spp. vector survival [1,3]. However, Vietnam now sees persistently high transmission baselines, recurring epidemic years, and dengue's geographic spread into both subtropical northern provinces and highland regions. Outbreaks in 2019, 2022, and 2024 revealed a structural change toward a warmer, more variable climate that supports long-term transmission, consistent with worldwide trends [1]. Climatic anomalies such as excessive heat, droughts, El Niño–Southern Oscillation (ENSO)-driven warming, and unpredictable rainfall now have greater impact on disease risk than long-term averages or historical baselines, highlighting the collapse of “stationary” epidemiology [2].

Consequently, Vietnam's traditional reliance on case-based endemic surveillance using five-year averages is increasingly unreliable [1,3]. Non-stationarity and baselines skewed by pandemic reporting or extreme outbreaks render thresholds misleading, risking both alert fatigue and missed warnings. This article summarizes growing research on Vietnamese climate–dengue interactions and advocates for a hybrid early warning system (EWS) combining operational climatic thresholds with probabilistic models. Routine surveillance should leverage accessible, biologically grounded indicators — including temperature and rainfall extremes — to guide targeted interventions.

While dengue virus does not circulate in animal reservoirs in the manner of classical zoonoses, a One Health framework remains essential because transmission is fundamentally governed by the health of the urban environment. Waste accumulation, inadequate sanitation, water storage practices driven by drought, and rapid urbanization that concentrates populations while straining infrastructure all create and sustain *Aedes* habitats [1,4]. Agricultural pesticide use further drives insecticide resistance, exemplifying environment–vector–human feedback loops that One Health addresses. This is critical as water storage practices, urban livestock habitats, and climate-sensitive biological controls like *Wolbachia* alter climate–dengue interactions and must be factored into next-generation forecasts.

## 2 Key climatic measures affecting dengue risk

To date, most robust findings in Vietnam focus on temperature, hydrological anomalies, and extreme occurrences. Dengue transmission is influenced not by broad seasonal trends, but by precise environmental thresholds affecting virus replication, vector competence, and breeding site availability. Table 1 summarizes key climatic thresholds with information for dengue early warning, acknowledging that climate–dengue relationships are non-linear, context-specific, and strongly modified by other factors. Importantly, these thresholds should not be applied uniformly across Vietnam's diverse climate gradient. In the South region, where mean temperature exceeds 27 °C year-round, temperature alone is not discriminatory; hydrological signals and ENSO phases serve as primary indicators. In Northern and Central regions, temperature and diurnal temperature range (DTR) thresholds retain strong predictive power for seasonal transmission onset [1,2].

**Table 1:** Climatic thresholds for dengue early warning in Vietnam.

Climatic variable	Threshold/condition	Direction of risk	Mechanism	Typical lead time	Region-specific applicability	Reference
Mean temperature	> 27 °C	Increases risk	Shortens EIP, increases feeding frequency	1–3 months	Year-round in South (use hydrological triggers instead); seasonal marker in North and Central	[1,5,6]
Minimum temperature	> 22 °C	Sustains transmission	Allows continuous mosquito activity and survival	1–3 months	Critical in North for overwintering survival; less discriminatory in South	[6]
DTR at low means	High DTR at low means (e.g., 20–24 °C)	Increases risk	Midday highs accelerate viral replication despite cool nights	2–6 weeks	Most relevant in North during cool season onset	[7]
DTR at high means	High DTR at high means (e.g., 28–30 °C)	Moderates risk	Temperature stress reduces survival and fecundity	Immediate	Applicable primarily in South during extreme heat	[7]
Heatwave	5–7 consecutive days with max daily temperature above the local 95th percentile	Delayed surge	Initial mortality; later population rebound	4–12 weeks	Relevant nationwide; delayed rebound most notable in South	[5]
Moderate rainfall	130–210 mm/week	Increases risk	Creates ephemeral container habitats	2–4 weeks	Key in South and Central; less informative in North dry season	[5,8]
Extreme rainfall	scPDSI ≈ +4 (extremely wet)	Short-term reduction	Larval flushing from containers	1–2 weeks	Most relevant in Central (typhoons) and South (monsoons)	[5,8]
Drought	scPDSI in the range of −3 to −4 (severe to extreme drought)	Substantial risk increase	Water storage in jars/tanks creates stable breeding sites	4–12 weeks	Highest impact in South and Central; dependent on urban water storage	[1,5]
Consecutive dry days	15–20 days	Increases risk	Proxy for drought; triggers household storage	2–8 weeks	Relevant in all regions; triggers water storage behavior	[1,5]
ENSO (El Niño phase)	NINO3.4 anomaly increases by 1 °C	Regional epidemic risk	Regional warming, synchronized epidemics	Seasonal (2–6 months)	Strongest effect in South and Central; weaker in far North	[2]

Note: Thresholds represent candidate indicators for early warning and should be calibrated using local epidemiological data. Uniform application is ineffective due to Vietnam's diverse climate gradient; in the consistently warm South, hydrological and ENSO signals provide the clearest warning, whereas thermal thresholds are critical in the North and Central regions. Abbreviations: DTR, diurnal temperature range; EIP, extrinsic incubation period; scPDSI, self-calibrated Palmer Drought Severity Index (indicative values: ≤ −3, severe drought; ≤ −4, extreme drought; +4, extremely wet conditions); ENSO, El Niño–Southern Oscillation; NINO3.4, sea surface temperature anomaly index for Central–Eastern Pacific (El Niño monitoring).

First, temperature remains the primary risk predictor [1,[5], [6], [7]]. A mean of 27 °C represents a biological tipping point, shortening the extrinsic incubation period to maximize infectivity [1,5,6]. Vietnam's Southern region exceeds this threshold throughout the year, explaining hyperendemic transmission; while in more northern areas it is gradually reached or exceeded in spring and summer as winters warm, allowing eggs and, occasionally, adult mosquitoes to overwinter. However, local predictive accuracy often requires monitoring minimum temperatures and humidity (i.e. urban microclimates) [8]. Additionally, DTR modulates risk [7]. Large DTRs can paradoxically raise transmission at lower mean temperatures by allowing midday highs to support viral replication, explaining why early-season dengue outbreaks in Hanoi occur during “cool” months [9].

Second, hydro-meteorological extremes provide further risk. Moderate rainfall (approximately 130–210 mm/week) promotes *Aedes* breeding, while intense precipitation temporarily reduces risk by flushing larvae from exposed habitats [5]. This typically occurs when rainfall exceeds container overflow, yet in urban areas, peri-domestic mosquito breeding sites are frequently indoor or semi-sheltered and therefore not flushable. Drought often exerts a more potent effect. The self-calibrated Palmer Drought Severity Index (scPDSI) identifies local anomalies where water scarcity prompts increased household storage in jars, buckets, and drums, creating stable breeding habitats immune to rainfall fluctuations [1,5]. While storage behaviors and piped-water availability vary by region and infrastructure reliability, this “drought paradox” — whereby dry conditions elevate risk — is well documented in Vietnam, frequently preceding outbreaks by one to three months.

Third, extreme weather and teleconnections, namely ENSO, serve as regional synchronizers. El Niño occurrences typically raise temperatures in Southeast Asia and Western Pacific regions, accounting for significant interannual fluctuation [2]. Heatwaves introduce further complexity: while prolonged heat may initially increase vector mortality, it frequently causes a delayed rebound, resulting in substantial epidemic peaks eight to twelve weeks later [5]. Recognizing this latency is critical to predicting late-season surges.

Critically, the differential lead times associated with each climatic variable (Table 1) have direct implications for EWS design. Temperature and drought signals offer the longest lead times (1–3 months), providing a strategic window for resource prepositioning and community engagement. Rainfall signals operate on shorter timescales (2–4 weeks), requiring rapid tactical responses. ENSO indicators provide a 2–6 months horizon but at lower spatial resolution [2], making them best suited to national-level planning. These differential lags inform tiered response protocols in which slow-onset signals activate preparedness and short-onset signals trigger immediate action.

## 3 Key predictive models and their practical use

Predicting dengue risk in Vietnam requires balancing scientific precision with practical utility. Three primary modeling approaches — probabilistic superensembles, endemic channels, and machine learning — offer distinct trade-offs for national and subnational applications.

The probabilistic superensemble is currently cutting-edge. By combining hundreds of sub-models based on climate signals and weighting them by past performance, it produces calibrated outputs superior to single models [1,3]. Superensembles enable greater epidemic detection with lead periods of one to three months, and they are particularly successful in the south of Vietnam, where seasonal signals are robust and climatic forcing is stable [1,3]. However, high computational demands and data requirements limit district-level accessibility. Furthermore, performance suffers during anomalous events like COVID-19 or genotype shifts. Consequently, superensembles are best suited for strategic planning (e.g., projecting seasonal peaks, budgeting, pesticide stockpiling) rather than local response.

Conversely, traditional endemic channels (“endemic curve”) remain the operational foundation due to their simplicity, triggering alerts when cases exceed the five-year average by two standard deviations [1,3]. However, this method is increasingly brittle. Major outbreaks inflate baselines, masking dangerous rises, while pandemic years lower them, causing false positives. As climatic non-stationarity increases, historical averages diverge from biological reality [1,3]. Moreover, reliance on clinical presentation may lead to significant underreporting, as a substantial proportion of dengue infections are asymptomatic — estimated at approximately 59% globally (95% confidence interval [*CI*]: 44%–75%), although reaching up to 80% during outbreak periods [10] — and these undetected carriers are critical to sustained transmission. Despite these limitations, endemic channels are useful for retrospective monitoring and communication, but only when separated from early-warning obligations.

A third category, machine learning models (MLMs) like random forests, extreme gradient boosting (XGBoost), and long short-term memory (LSTM) networks, has gained popularity in Vietnam [3,11]. These excel at capturing non-linear interactions and theoretically would be better suited to climate-urbanization couplings than typical regression-based systems. Data mining approaches to predict dengue outbreaks have been applied with some success in other countries with complex climate variables [12]. However, MLMs require large, clean datasets and computer science skills to use, while their interpretability is still limited. Without proper operationalization, they risk becoming intellectual exercises rather than useful tools. Their most promising addition is feature identification, which reveals, for example, that minimum temperature and population density consistently outperform mean temperature at prediction accuracy.

A practical consideration for operationalizing any EWS is the choice of climate data source and spatial resolution. Ground-based meteorological stations provide high temporal resolution but suffer from spatial gaps, particularly in mountainous and rural Vietnam. Gridded reanalysis and remotely sensed products (e.g., ECMWF Reanalysis v5 [ERA5], Climate Hazards Group InfraRed Precipitation with Station [CHIRPS] data, Moderate Resolution Imaging Spectroradiometer [MODIS]) offer spatially continuous coverage but may smooth urban microclimatic variation critical to *Aedes* ecology. By consensus, satellite-derived data suit national and provincial models, while district-level tactical triggers benefit from local station data calibrated against epidemiological ground truth. Table S1 in supplementary material details the requirements, merits, and applicability of these approaches.

## 4 Policy and operations recommendations for Vietnam's next-generation EWS

Modernizing dengue early warning in Vietnam requires a change from reactive, case-based surveillance to proactive, climate-sensitive triggers that are anchored in a One Health framework. Three governing principles are outlined below.

First, a climatic trigger-based surveillance system should supplement, not replace, existing endemic channels. Triggers must be location-specific: in the Northern region, temperature and DTR thresholds are more informative; while further south, hydrological signals and ENSO anomalies take precedence. Crucially, triggers must remain accessible and understandable to district-level officers, facilitating rapid and proportionate responses independent of sophisticated modeling results. A legitimate concern with any threshold-based system is the risk of low specificity generating alert fatigue among district health officers. The proposed hybrid system mitigates this through several design features: graduated responses, where initial threshold crossings activate low-cost, “no regrets” actions (e.g., community mobilization, larval surveillance) rather than full emergency responses; convergent signaling, where concurrent crossing of multiple indicators escalates the alert level, substantially improving specificity; and regional calibration, ensuring triggers reflect local epidemiological context rather than national averages [1,[5], [6], [7]]. Case-based surveillance data provide confirmatory evidence, creating a two-stage verification process that balances sensitivity with specificity.

Second, as Vietnam is 1650 km in length and has a land area of 330,000 km^2^, geographic stratification is necessary. The nation's climate gradient means that risk drivers range significantly by location, especially affected by latitude and altitude. Dengue risk in the Southern region is increasingly informed by rain–drought cycles, water storage habits, and major human migration fluxes. Overwintering eggs, urban heat islands, and extreme rainfall patterns are progressively shaping risk in the Northern region, blurring traditional seasonal boundaries [1,4,5]. Central provinces serve as critical transition zones for ENSO and typhoon activity [2]. A national EWS that ignores these distinctions threatens misallocating resources or making projections that are broadly representative but imprecise locally.

Third, Vietnam should integrate One Health concepts directly into EWS design. Urban and peri-urban livestock farming creates breeding habitats that sustain vector populations outside household settings. While *Aedes aegypti* is strongly anthropophilic, the link between farming and dengue risk operates primarily through environmental pathways: livestock operations maintain uncovered water troughs, drums, and storage containers that serve as larval habitats. These also support *Aedes albopictus*, a secondary but increasingly important dengue vector with a broader ecological niche [1,8]. Additionally, biological controls require climatic monitoring; for instance, heat stress can compromise *Wolbachia* density, impairing dengue transmission-blocking efficiency. Similarly, agriculturally driven insecticide resistance should dictate the viability of chemical controls during outbreaks. Incorporating these biological and environmental feedback loops into surveillance algorithms ensures that climatic triggers prompt context-appropriate responses rather than uniform, often ineffectual, activities.

Fundamentally, the health system should consider climatic events as signals for readiness rather than as predictions. When spring temperatures exceed suitability limits early, droughts worsen, or heatwaves occur, Vietnam gains a window of opportunity to act. Taking responsibility to leverage this lead time must be institutionalized by preparing standard operating procedures that stipulate under which circumstances specified initiatives are activated, which units are accountable, and what timelines are expected. After all, even the most accurate prognosis is rendered ineffective in the absence of operational clarity.

## 5 Conclusion

The incidence and distribution of dengue in Vietnam are increasingly characterized by climate volatility rather than predictable seasonality. While advanced probabilistic models provide valuable national-scale insights, the future operational core of Vietnam's EWS should be based on easily accessible climatic thresholds that encode thermal, hydrological, and extreme-event sensitivities of *Aedes* mosquito vectors and dengue virus replication. A hybrid system: superensembles for strategic forecasting, thresholds for tactical readiness, and One Health integration for contextual accuracy, provides the most feasible path forward. This method reframes early warning as anticipatory action based on environmental suitability, rather than case prediction. In a rapidly changing climate where dengue risk is escalating, aligning public health interventions with environmental signals may enable Vietnam to transition from reacting to outbreaks to preemptively reducing their scale and impact.

## CRediT authorship contribution statement

**Nguyen Khoi Quan:** Writing – original draft, Investigation, Formal analysis, Conceptualization. **Andrew W. Taylor-Robinson:** Writing – review & editing, Validation, Supervision, Conceptualization.

## Funding

This study received financial support from 10.13039/100032892VinUniversity (grant number VUNI.2324.SG02).

## Declaration of interests

The authors declare that they have no known competing financial interests or personal relationships that could have appeared to influence the work reported in this paper.
