# Supplementary material for: “Climatic determinants” and simple thresholds for dengue early warning in Vietnam: a One Health perspective
Source: Sci One Health. 2026 Apr 28;5:100159. doi: 10.1016/j.soh.2026.100159 (PMC13202247; doi:10.1016/j.soh.2026.100159)
Supplement: Multimedia component 1 [file mmc1.docx]

**Supplementary Material**

**Table S1. Comparison of predictive approaches in Vietnam.**

| **Approach** | **Inputs required** | **Strengths** | **Limitations** | **Best operational level** |
| --- | --- | --- | --- | --- |
| **Probabilistic superensemble models** | Temperature (minimum/maximum/mean), humidity, rainfall, DTR, ENSO indices, land cover | Highest predictive skill at 1–3 months; quantifies uncertainty; strong for resource planning | Requires computation & expertise; degraded performance in atypical years; limited district usability | National & Provincial (strategic planning) |
| **Machine learning models** (random forest, XGBoost, LSTM neural network) | Climate + demographic + mobility data | Captures nonlinear interactions; helpful for feature discovery | Interpretability low; requires staff training; rarely operationalized | National/research |
| **Statistical regression models** | Climate + lagged cases | Simple, interpretable; low resource demand | Sensitive to non-stationarity; less robust under extremes | Provincial/some district use |
| **Case-based endemic channels** (classic) | Surveillance case counts (5-year baseline) | Familiar to local staff; quick; intuitive | Reactive; thresholds distorted by outbreak or COVID years | District (retrospective monitoring only) |
| **Threshold-based triggers** (the proposed hybrid system) | Temperature, rainfall, drought index, heatwave duration | Simple, fast, robust to missing case data; pre-emptive | Requires calibration & clear SOPs | District & provincial (tactical response) |

Abbreviations: EWS, Early Warning System; XGBoost, extreme gradient boosting; LSTM, long short-term memory; SOP, standard operating procedure.
